# Time- and Region-specific Effect of Vortioxetine on Central LPS-induced Transcriptional Regulation of NLRP3 Inflammasome

**DOI:** 10.2174/1570159X22666240705143649

**Published:** 2024-07-12

**Authors:** Miriam Ciani, Giovanna Rigillo, Cristina Benatti, Luca Pani, Johanna M.C. Blom, Nicoletta Brunello, Fabio Tascedda, Silvia Alboni

**Affiliations:** 1 Department of Life Sciences, University of Modena and Reggio Emilia, Modena, Italy;; 2 Department of Biomedical, Metabolic and Neural Sciences, University of Modena and Reggio Emilia, Modena, Italy;; 3 Centre of Neuroscience and Neurotechnology, University of Modena and Reggio Emilia, Modena, Italy;; 4 Department of Psychiatry and Behavioral Sciences, University of Miami, Miami, USA;; 5 CIB, Consorzio Interuniversitario Biotecnologie, Trieste, Italy

**Keywords:** Depression, NLRP3 inflammasome, vortioxetine, cognitive impairment, neuroinflammation, hippocampus, frontalprefrontal cortex, hypothalamus

## Abstract

**Background:**

Inflammasome overactivation, multiprotein complexes that trigger inflammatory responses, plays a critical role in Major Depressive Disorder (MDD) pathogenesis and treatment responses. Indeed, different antidepressants alleviate depression-related behaviours by specifically counteracting the NLRP3 inflammasome signalling pathway. The immunomodulatory effects of vortioxetine (VTX), a multimodal antidepressant with cognitive benefits, were recently revealed to counter memory impairment induced by a peripheral lipopolysaccharide (LPS) injection 24 hours (h) post-challenge. The potential link between VTX and NLRP3, along with other inflammasomes, remains un-explored.

**Methods:**

The potential link between VTX and NLRP3, along with other inflammasomes, remains unexplored. Hence, adult C57BL/6J male mice (n = 73) were fed with a standard or VTX-enriched diet (600 mg/kg of food, 28 days), injected with LPS (830 μg/kg) or saline, and sacrificed 6/24 h post-LPS. At these time-points, transcriptional effects of LPS and VTX on NLRP3, NLRP1, NLRC4, AIM2 (inflammasomes), ASC and CASP1 (related subunits) and NEK7 mediator (NLRP3 regulator) were assessed in dorsal and ventral hippocampal subregions, frontal-prefrontal cortex and hypothalamus, brain regions serving behavioural-cognitive functions impaired in MDD.

**Results:**

Varied expression patterns of inflammasomes were revealed, with long-term NLRP3 and ASC transcriptional changes observed in response to LPS. It was demonstrated that VTX counteracted the LPS-mediated NLRP3 and ASC upregulation in memory-related brain areas like the dorsal hippocampus at 24 h time-point, potentially *via* regulating NEK7 expression. No VTX-mediated transcriptional effects were observed on other inflammasomes, reinforcing a potentially specific modulation on the NLRP3 inflammasome signalling pathway.

**Conclusion:**

Thus, a novel VTX molecular mechanism in modulating the NLRP3 inflammasome in a time- and area-specific manner in the brain was highlighted, with significant clinical implications in treating depression and cognitive impairments.

## 1 INTRODUCTION

Major depressive disorder (MDD) is a significant public health problem that profoundly impacts individuals and their families, urging targeted therapies due to its escalating global prevalence, particularly in vulnerable stages like adolescence [1, 2]. Existing antidepressants have limitations, especially in addressing the MDD’s complex and heterogeneous nature encompassing emotional, cognitive, and behavioral symptoms [3, 4]. Of note, cognitive impairments persist despite mood improvement [5], prompting the search for treatments with pro-cognitive effects, yet the underlying molecular mechanisms remain inadequately understood [6]. Persistent inflammatory and immune dysregulations are pivotal in MDD, influencing treatment outcomes [7]. Inflammasomes, crucial in triggering inflammatory responses, have garnered attention in MDD pathogenesis [8-14]. These multimeric protein complexes typically comprise three subunits: a specific sensor detecting harmful stimuli, the apoptosis-associated speck-like protein (ASC) bridging other players *via* distinct domains, and caspase-1 (CASP1) essential for interleukin (IL)-1β and IL-18 maturation, determining factors in processes that include cognition and memory [15, 16]. The NOD-like receptor family pyrin domain containing (NLRP) 3, NLRP1, the NLR family CARD domain containing (NLRC) 4, and the absence in melanoma-2 (AIM2), classified by sensor proteins, are implicated in brain functions and related diseases, including MDD. Their regulated activation aids tissue repair, yet dysregulation can lead to pathology [17-23]. Notably, NLRP3 inflammasome-mediated neuroinflammation is extensively studied in MDD, with increased activation observed in depressive models and patients [9-11, 14]. Certain antidepressants alleviate depression-related behavior by counteracting NLRP3 activation [8, 11, 13, 24]. However, understanding NLRP3’s contribution in leading different components of disease, particularly in mediating depression-related cognitive impairments and their response to targeted treatment, remains an ongoing investigation [25-29]. Vortioxetine (VTX), a recent multimodal antidepressant, has progressively stood out in treating various neurodegenerative and psychiatric conditions for its cognitive benefits [30-32]. Preclinical and clinical studies reveal its effectiveness in alleviating depressive symptoms and enhancing memory, cognition, and executive functions, even preventing depression relapse [33, 34]. Additionally, VTX’s immunomodulatory effects were recently demonstrated, specifically countering memory impairment induced by a peripheral lipopolysaccharide (LPS) injection 24 hours (h) after the immune challenge [35]. Despite these observations, the potential link between VTX and NLRP3 inflammasome, along with other inflammasomes, remains unexplored. To bridge this gap, by using an LPS-induced depression-like animal model, we conducted a thorough gene expression analysis of crucial inflammasomes (NLRP3, NLRP1, NLRC4, AIM2) and related subunits (ASC and CASP1) in specific cortical-subcortical brain regions involved in behavioral-cognitive functions impaired in depression, assessing the effects of a chronic VTX pre-treatment on these genes at two different time-points post-LPS injection. Our hypotheses centered on differential inflammasome expression patterns in response to LPS across brain regions. We anticipated VTX’s impact on LPS-induced NLRP3 inflammasome expression primarily in memory-related brain areas. Additionally, as the never-in-mitosis gene a (NIMA)-related expressed kinase 7 (NEK7) mediates NLRP3 inflammasome activation [36], NEK7 expression was also assessed to explore a potential VTX mechanism of action in modulating NLRP3 expression.

## 2 MATERIALS AND METHODS

### 2.1 Animals

Adult C57BL/6J male mice 11-16 weeks old (n = 73; Charles River Laboratories, Lecco, Italy) were randomly group-housed in polycarbonate cages (four per cage of 30 × 30 × 15 cm) and habituated for five weeks prior to pharmacological treatment with free access to water and food, under a 12:12 light-dark cycle (lights on 8:00 am to 8:00 pm) at a room temperature of 21 ± 3°C with relative controlled humidity. Throughout the experiment, for habituation to the experimenters, animals were handled twice a week and checked daily for signs of discomfort (animal care and use guidelines National Academy of Sciences. Guide for the Care and Use of Laboratory Animals, 1998, “Guidelines for the Care and Use of Mammals in Neuroscience and Behavioral Research” (National Research Council 2003)). All procedures were carried out in accordance with the EC guidelines (EEC Council Directive 86/609 1987) and the Italian legislation on animal experimentation (Decreto Legislativo 26/2014) and had the approval of the local Ethical Committee.

### 2.2 Treatment

**Fig. (1):**
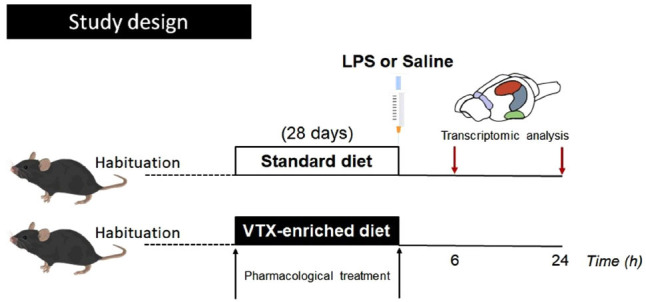
Schematic representation of THE experimental design. After habituation, adult C57BL/6J male mice (n = 73) were fed for 28 days with a standard (n = 37) or a VTX-enriched diet (600 mg base per kg of food) (n = 36) before being injected with lipopolysaccharide (LPS, 830 μg/kg) (n = 37) or the same volume of vehicle (pyrogen-free saline) (n = 36). At two different time points after injection (6 and 24 h, hours), animals were sacrificed for multi-central area gene expression analyses of the inflammasomes and related subunits. **Abbreviations:** vortioxetine, VTX; lipopolysaccharide, LPS; hours, h.

For 28 days, mice were fed with a standard diet based on Purina 5001 rodent chow or a VTX-enriched diet with Purina 5001 rodent chow containing VTX (synthesized by H. Lundbeck A/S, Valby, Denmark) at a concentration of 600 mg base per kg food (Research Diets Inc., New Brunswick, NJ). Animals had access to plain tap water ad libitum. The concentration of VTX was established to achieve the therapeutic dose range based on brain SERT occupancy [35, 37]. On day 29, the vehicle (pyrogen-free saline) or LPS from *Escherichia coli* (830 μg/kg; serotype 0127: B8, Sigma-Aldrich, St. Louis, MO, United States) were injected intraperitoneally. Six and twenty-four hours (6 h; 24 h) after the intraperitoneal treatment, mice were sacrificed, brains were removed, and tissues of interest were isolated and processed for the subsequent gene expression analysis. For habituation to the treatment procedure, mice were handled two days before the injection (Fig. **1**). Animals were weighed once a week and both 6 h/24 h before and after LPS/vehicle administration. The food and water intake of each cage were assessed twice a week. No significant changes in mean body weight and animal feeding and drinking were revealed [35].

### 2.3 Gene Expression Analyses

Total mRNA was extracted from dorsal (DH) and ventral (VH) hippocampal subregions, frontal-prefrontal cortex (FCx), and hypothalamus (HYP). Total RNA extraction and DNAse treatment were performed as previously described [35] using Gen Elute™ Mammalian Total RNA Miniprep Kit and DNASE 70-on Column DNase I Digestion Set (MerckKGaA, Germany). One microgram of total RNA was reverse transcribed with a High-Capacity cDNA Reverse Transcription Kit (Thermo Fisher Scientific, MA), and RT-qPCR was performed, as previously described [38], in CFX Connect Real-Time PCR machine (Bio-Rad Laboratories, CA) using SsoAdvanced Universal SYBR Green Supermix (Bio-Rad Laboratories, CA). Specifically, we evaluated NLRP3, NRLP1, NRLC4, AIM2, ASC, CASP1, and NEK7 gene expression by using specific forward and reverse primers at a final concentration of 300 nM (Table **1**). Data were normalized to the expression of the endogenous cyclophilin A (CypA), and the relative expression level of target genes was defined by the 2^-ΔΔCt^ method.

### 2.4 Statistical Analyses

Data are presented as mean ± standard error of the mean (SEM). All statistical analyses were performed using SPSS software version 28.0 (IBM Corp., Armonk, NY, USA). Molecular data were analyzed with two-way analysis of variance (ANOVA) for the main effects of LPS/vehicle treatment, VTX/standard diet, or interaction between them (LPS*x*VTX); planned pairwise comparisons were performed using one-way ANOVA followed by Tukey’s post-hoc test (LPS *vs*. vehicle animals receiving the same diet for four weeks or standard *vs*. VTX-enriched diet in mice injected with either LPS or vehicle) as previously described [35]. Extreme outliers were excluded prior to using the boxplot tool in SPSS (more than 3x the interquartile range outside of the end of the interquartile box). A *p*-value below 0.05 was considered statistically significant. Graph generation was conducted using GraphPad Prism 9 (GraphPad Software San Diego, CA, USA).

## 3 RESULTS

### 3.1 Effect of Vortioxetine on LPS-induced Transcriptional Regulation of NLRP3 Inflammasome and -related Subunits in the Interconnected Brain Regions Involved in Cognitive, Emotional, and Physiological Functions

#### 3.1.1 A 4-week Vortioxetine Administration Counteracted the LPS-induced NLRP3 Inflammasome and -related Subunits Transcription in the Dorsal and Ventral Hippocampal Subregions 24 h after Immune Challenge

**Fig. (2):**
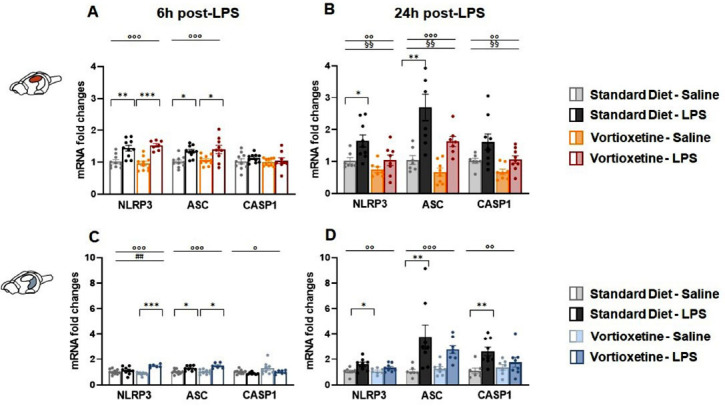
Vortioxetine pre-treatment counteracted both NLRP3 and ASC transcriptional upregulation induced by LPS 24 h-after-injection in the dorsal hippocampus. Bar graphs represent the mRNA expression levels of NLRP3 inflammasome subunits (NLRP3, ASC, and CASP1) at 6 h and 24 h post-LPS in the (**A**, **B**) dorsal (DH) and (**C**, **D**) ventral (VH) hippocampus. Data are shown as means ± SEM; n = 8-10 mice per group. Two-way ANOVA: ◦*p* < 0.05, ◦◦*p* < 0.01, ◦◦◦*p* < 0.001 main effect LPS/vehicle; §§*p* < 0.01 main effect standard/VTX-enriched diet; ##*p* < 0.001 main effect LPS*x*diet. Post-hoc: *p < 0.05, **p < 0.01, ***p < 0.001 indicate significant group differences). **Abbreviations:** NLR family, pyrin domain containing 3, NLRP3; apoptosis-associated speck-like protein, ASC; caspase 1, CASP1; dorsal hippocampus, DH; ventral hippocampus, VH; lipopolysaccharide, LPS; hours, h.

In the DH, the immune challenge significantly affected NLRP3 and ASC gene expression at both 6 h (NLRP3: F (1,36) = 46.538, *p <* 0.0001; ASC: F (1,38) = 19.459, *p <* 0.0001) and 24 h (NLRP3: F (1,30) = 8.946, *p =* 0.006; ASC: F (1,29) = 26.751, *p <* 0.0001) post-LPS injection (Figs. **2A**, **B**). However, for CASP1, exposure to LPS resulted in significant change only at 24 h post-injection (F (1,32) = 9.434, *p =* 0.005) (Fig. **2B**). Diet also had a significant impact, affecting all three targets at 24 h (NLRP3: F (1,30) = 8.328, *p =* 0.008; ASC: F (1,29) = 8.221, *p =* 0.008; CASP1: F (1,32) = 7.972, *p =* 0.009) (Fig. **2B**). Post-hoc analysis showed that, at 6 h post-LPS injection, both NLRP3 and ASC mRNA expression significantly increased regardless of diet (Fig. **2A**). However, at 24 h, this increase was observed only in mice fed with a standard diet compared to their non-LPS injected counterparts (Fig. **2B**). For CASP1, there were no significant differences in its expression within groups over time (Figs. **2A**,**B**). In the VH, all the three considered NLRP3 inflammasome subunits were significantly influenced by LPS exposure at both 6 h (NLRP3: F (1,33) = 25.444, *p <* 0.0001; ASC: F (1,33) = 22.991, *p <* 0.0001; CASP1: F (1,31) = 4.330, *p =* 0.047) and 24 h time points (NLRP3 24 h: F (1,29) = 10.664, *p =* 0.003; ASC 24 h: F (1,30) = 15.548, *p =* 0.001; CASP1 24 h: F (1,30) = 9.254, *p =* 0.005) (Figs. **2C**, **D**). Moreover, two-way ANOVA revealed an interaction between LPS and diet only for NLRP3 expression 6 h after the immune challenge (F (1,33) = 12.869, *p =* 0.001) (Figs. **2C**, **D**). Further analysis showed that 6 h after treatment, exposure to LPS resulted in a significant increase in NLRP3 mRNA levels in mice chronically pre-treated with VTX while moderately inducing ASC mRNA expression regardless of the diet. At the same time point, CASP1 transcription was not influenced by the immune challenge (Fig. **2C**). At 24 h post-LPS, NLRP3, ASC, and CASP1 mRNA levels were increased in mice fed with the standard diet, but not in animals receiving VTX-based diet (Fig. **2D**).

#### 3.1.2 The Transcriptional Regulation of NLRP3 Inflammasome Induced by LPS was not Affected by Vortioxetine Administration in the Frontal-prefrontal Cortex and Hypothalamus

**Fig. (3):**
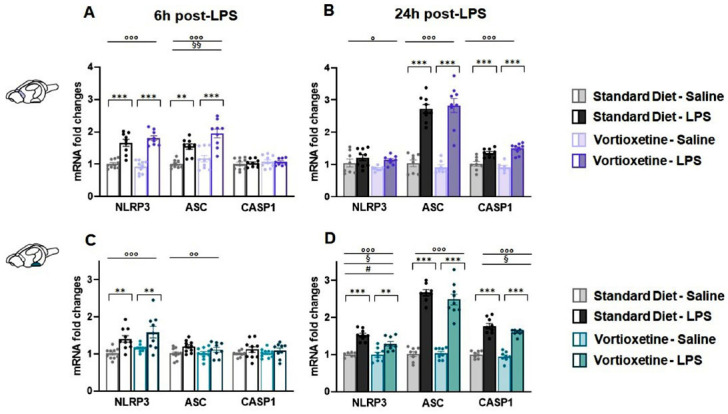
Vortioxetine pre-treatment did not modulate the LPS-induced transcriptional effects on the three considered NLRP3 inflammasome-related targets in the frontal-prefrontal cortex and in the hypothalamus. Bar graphs represent the mRNA levels of NLRP3, ASC, and CASP1 at 6 h and 24 h post-LPS in the (**A**, **B**) frontal-prefrontal cortex (FCx) and (**C**, **D**) hypothalamus (HYP). Data are reported as means ± SEM; n = 8-10 mice per group. Two-way ANOVA: ◦*p* < 0.05, ◦◦*p* < 0.01, ◦◦◦*p* < 0.001 main effect LPS/vehicle; §*p* < 0.05, §§*p* < 0.01 main effect standard/VTX-enriched diet; # *p* < 0.05 main effect LPS*x*diet. Post-hoc: **p < 0.01, ***p < 0.001 indicate significant group differences). **Abbreviations:** NLR family, pyrin domain containing 3, NLRP3; apoptosis-associated speck-like protein, ASC; caspase 1, CASP1; frontal-prefrontal cortex, FCx; hypothalamus, HYP; lipopolysaccharide, LPS; hours, h.

In FCx, the immune challenge significantly altered NLRP3 and ASC expression at both 6 h (NLRP3: F (1,37) = 112.738, *p <* 0.0001; ASC: F (1,36) = 47.408, *p <* 0.0001) and 24 h (NLRP3: F (1,31) = 6.318, *p =* 0.018; ASC: F (1,32) = 140.314, *p <* 0.0001) (Figs. **3A**, **B**). Exposure to LPS induced a significant change in CASP1 transcription only at 24 h post-injection (F (1,32) = 64.357, *p <* 0.0001) (Fig. **3B**). In addition, two-way ANOVA revealed a main effect of VTX pre-treatment on ASC expression 6 h-post LPS injection (F (1,36) = 8.504, *p =* 0.006) (Fig. **3A**). Post-hoc analysis indicated that, irrespective of the diet received, the mRNA levels of all the NLRP3 inflammasome subunits were affected by the immune challenge in a time-dependent way. In particular, the transcriptional NLRP3 upregulation was observed 6 h following LPS injection and returned to control levels at the 24 h time-point (Figs. **3A**, **B**), while ASC mRNA levels were already upregulated at 6 h and continued to increase 24 h post-LPS in both standard and VTX-enriched diet animals (Figs. **3A**, **B**). Finally, a significant increase in CASP1 mRNA levels was observed only 24 h after the immune challenge (Figs. **3A**, **B**).

In the HYP, as observed for all the other central areas considered, the immune challenge significantly affected NLRP3 and ASC expression at both 6 h (NLRP3: F (1,38) = 21.452, *p <* 0.0001; ASC: F (1,38) = 7.814, *p =* 0.008) and 24 h (NLRP3: F (1,32) = 56.088, *p <* 0.0001; ASC: F (1,32) = 250.615, *p <* 0.0001) (Figs. **3C**, **D**). Again, the immune challenge altered CASP1 transcription only at the 24-hour time-point (F (1,32) = 194.211, *p <* 0.0001) (Fig. **3D**). Diet also had a significant impact on the expression of NLRP3 (F (1,32) = 4.750, *p =* 0.038) and CASP1 (F (1,32) = 4.637, *p =* 0.040) 24 h post-LPS treatment. In addition, two-way ANOVA revealed an interaction between LPS and diet for NLRP3 transcription 24 h after immune challenge injection (F (1,32) = 4.452, *p =* 0.040) (Fig. **3C**). Similar to what was observed in the FCx, post-hoc analysis showed that in the HYP, the expression of the NLRP3 inflammasome subunits changed over time following the immune challenge but was not affected by the diet received. Indeed, within the LPS-treated groups, NLRP3 mRNA levels significantly increased over the first 6 h and remained higher up to 24 h. On the contrary, the immune challenge markedly induced ASC and CASP1 transcriptional upregulation only at the latter time-point (Figs. **3C**, **D**).

### 3.2 Vortioxetine and LPS Modulated the Transcription of NLRP1, NLRC4, AND AIM2 Inflammasomes in Brain Regions Involved in Cognitive, Emotional, and Physiological Functions

#### 3.2.1 Vortioxetine and LPS Differently Regulated the Expression of NLRP1, NLRC4, AND AIM2 Inflammasomes in the Dorsal and Ventral Hippocampal Subregions without an Interaction between the Two Factors

**Fig. (4):**
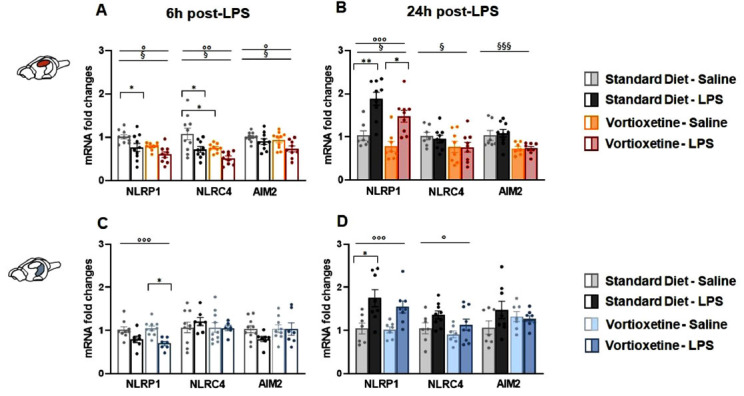
Vortioxetine selectively modulated the mRNA levels of NLRP1, NLRC4, and AIM2 inflammasomes in the dorsal hippocampus without affecting their expression in the ventral hippocampus. Bar graphs represent the transcriptional expression of NLRP1, NLRC4, and AIM2 inflammasomes at 6 h and 24 h post-LPS in the (**A**, **B**) dorsal (DH) and (**C**, **D**) ventral (VH) hippocampus. Data are reported as means ± SEM; n = 8-10 mice per group. Two-way ANOVA: ◦*p* < 0.05, ◦◦*p* < 0.01, ◦◦◦*p* < 0.001 main effect LPS/vehicle; §*p* < 0.05, §§§*p* < 0.001 main effect standard/VTX-enriched diet. Post-hoc: *p < 0.05, **p < 0.01 indicate significant group differences). **Abbreviations:** NLR family pyrin domain containing 1, NLRP1; NLR family, CARD domain containing 4, NLRC4; absent in melanoma 2, AIM2; dorsal hippocampus, DH; ventral hippocampus, VH; lipopolysaccharide, LPS; hours, h.

In the DH, the immune challenge significantly altered the expression of all the three considered inflammasomes at 6 h (NLRP1: F (1,37) = 11.158, *p =* 0.002; NLRC4: F (1,36) = 12.146, *p <* 0.0001; AIM2: F (1,38) = 7.156, *p =* 0.011) and only of NLRP1 at 24 h after treatment (F (1,37) = 33.964, *p <* 0.0001) (Figs. **4A**, **B**). Moreover, two-way ANOVA revealed that diet had a significant impact affecting all three inflammasomes at both 6 h (NLRP1: F (1,37) = 9.265, *p =* 0.004; NLRC4 (F (1,36) = 9.616, *p =* 0.004; AIM2 (F (1,38) = 4.542, *p =* 0.040) and 24 h (NLRP1: F (1,32) = 6.227, *p =* 0.019; NLRC4: F (1,32) = 4.347, *p =* 0.046; AIM2: F (1,30) = 16.232, *p <* 0.0001) (Figs. **4A**, **B**). Post-hoc analysis showed that the immune challenge decreased NLRP1 mRNA 6 h after treatment in control mice (Fig. **4A**); at 24 h, the expression of this target was significantly upregulated by LPS with respect to their saline-receiving counterparts in both standard and VTX-enriched diet animals (Fig. **4B**). Moreover, 6 h after the immune challenge, NLRC4 mRNA levels were decreased by LPS in animals fed with a standard diet compared to their vehicle-matching controls (Fig. **4A**). At the same time-point, saline-injected mice chronically fed with a VTX-enriched diet showed lower levels of NLRC4 expression compared to their standard diet-receiving counterparts (Fig. **4A**). There were no significant differences in AIM2 gene expression between groups at any of tested time-points (Figs. **4A**, **B**).

In the VH, two-way ANOVA revealed a main effect of LPS treatment at both time points on gene expression of NLRP1 (6 h: F (1,32) = 20.532, *p <* 0.0001; 24 h: F (1,29) = 17.559, *p <* 0.0001) and of NLRC4 only after 24 h (F (1,31) = 5.591, *p =* 0.025). According to post-hoc analysis, LPS induced a significant decrease in NLRP1 expression in animals chronically pre-treated with VTX compared their vehicle-receiving controls at the 6 h time-point (Fig. **4C**). Instead, an NLRP1 mRNA upregulation was observed 24 h after the immune challenge only in animals fed with a standard diet (Fig. **4D**). There were no significant differences in NLRC4 and AIM2 gene expression between groups at any of tested time-points (Figs. **4C**, **D**).

#### 3.2.2 A 4-week Pre-treatment with Vortioxetine and LPS Induced Similar Effects on the Transcription of NLRP1, NLRC4, and AIM2 Inflammasomes in the Frontal-prefrontal Cortex and Hypothalamus

**Fig. (5):**
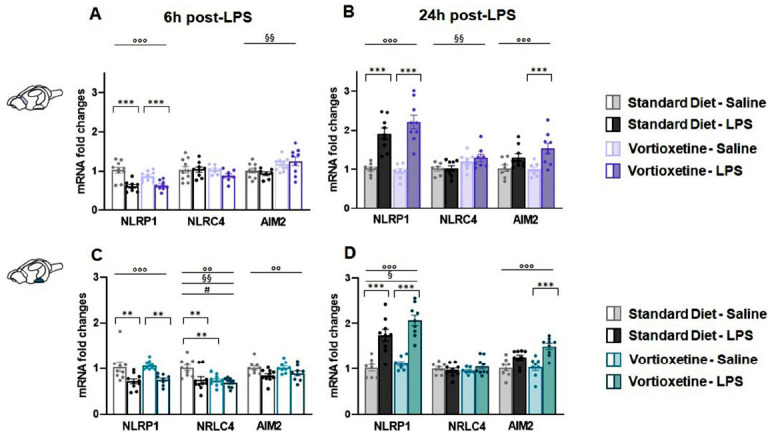
Transcriptional regulation of NLRP1, NLRC4, and AIM2 inflammasomes was similarly modulated following a VTX-enriched diet and LPS injection in the frontal-prefrontal cortex and hypothalamus. Bar graphs represent the mRNA levels of NLRP1, NLRC4, and AIM2 at 6 h and 24 h post-LPS in the (**A**, **B**) frontal-prefrontal cortex (FCx) and (**C**, **D**) hypothalamus (HYP). Data are reported as means ± SEM; n = 8-10 mice per group. Two-way ANOVA: ◦◦*p* < 0.01, ◦◦◦*p* < 0.001 main effect LPS/vehicle; §*p* < 0.05, §§*p* < 0.01 main effect standard/VTX-enriched diet. Post-hoc: **p < 0.01, ***p < 0.001 indicate significant group differences). **Abbreviations:** NLR family pyrin domain containing 1, NLRP1; NLR family, CARD domain containing 4, NLRC4; absent in melanoma 2, AIM2; frontal-prefrontal cortex, FCx; hypothalamus, HYP; lipopolysaccharide, LPS; hours, h.

In the FCx, exposure to LPS significantly altered expression at both time points (6 h: F (1,37) = 38.898, *p <* 0.0001; 24 h: F (1,32) = 69.677, *p <* 0.0001) and AIM2 only at 24 h (F (1,32) = 15.741, *p <* 0.0001) (Figs. **5A**, **B**). In addition, two-way ANOVA evidenced a significant main effect of the diet on the transcription of AIM2 at 6 h (F (1,34) = 7.303, *p =* 0.011) and NLRC4 at 24 h (F (1,30) = 8.643, *p =* 0.007) (Figs. **5A**, **B**). Post-hoc analysis showed that the immune challenge caused a significant reduction of NLRP1 mRNA levels 6 h after treatment (Fig. **5A**) while markedly enhancing the expression of this target at the latter time point in animals fed with standard or VTX-enriched diet (Fig. **5B**). A significant increase in AIM2 mRNA levels was observed in LPS-exposed animals pretreated with VTX with respect to their matching controls at the 24 h time-point (Fig. **5B**).

In the HYP, LPS injection significantly altered the expression of all the three genes at 6 h (NLRP1: F (1,37) = 26.151, *p <* 0.0001; NLRC4: F (1,38) = 8.855, *p =* 0.005; AIM2: F (1,36) = 10.427, *p =* 0.003), while 24 h post-injection this effect was observed only for NLRP1 (F (1,33) = 70.585, *p <* 0.0001) and AIM2 (F (1,33) = 27.653, *p <* 0.0001). The diet also had a significant impact, influencing NLRC4 at the 6 h time-point and NLRP1 at 24 h (F (1,33) = 4.358, *p =* 0.045). Two-way ANOVA also revealed an interaction among the factors (VTX*x*LPS) for the expression of NLRC4 (F (1,38) = 4.484, *p =* 0.041) at the earliest time-point assessed (Figs. **5C**, **D**). Post-hoc analysis showed that LPS injection affected NLRP1 and AIM2 transcription with a pattern similar to that observed in the FCx. Specifically, the immune challenge reduced NLRP1 expression 6 h after treatment while markedly enhancing its expression at the latter time point in animals fed with both standard or VTX-enriched diets (Figs. **5C**, **D**). Moreover, a moderate increase in AIM2 mRNA levels was observed in LPS-exposed animals pretreated with VTX with respect to their matching controls at the 24 h time-point (Fig. **5D**). As for NLRC4 gene, at the 6 h time point, its expression was reduced in standard diet-fed animals injected with LPS compared to their vehicle-exposed counterparts; at the same time-point, exposure to a VTX-enriched diet resulted in lower levels of this target with respect to animals fed with standard diet (Fig. **5C**).

### 3.3 Vortioxetine and LPS Modulated the Transcription of NEK7 in Brain Regions Involved in Cognitive, Emotional, and Physiological Functions

#### 3.3.1 A 4-week Pre-treatment with VTX Reduced the Expression of NEK7 in the Dorsal Hippocampus 24 h after an LPS Injection

**Fig. (6):**
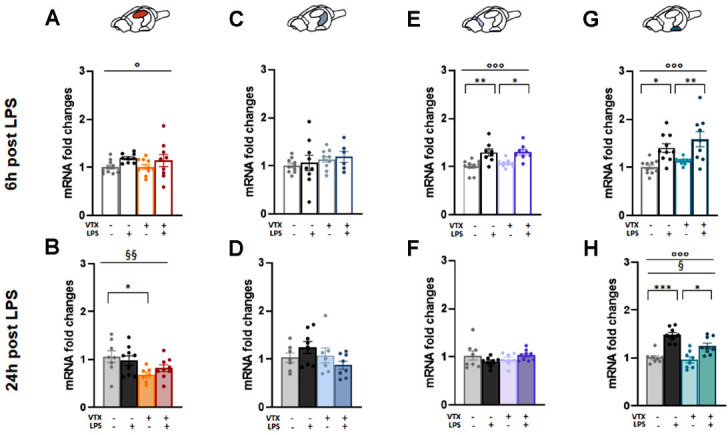
In the dorsal hippocampus, vortioxetine downregulated NEK7 expression. Bar graphs represent the mRNA levels of NEK7 at 6 h and 24 h post-LPS in the (**A**, **B**) dorsal hippocampus (DH), (**C**, **D**) ventral hippocampus (VH), (**E**, **F**) frontal-prefrontal cortex (FCx), (**G**, **H**) hypothalamus (HYP). Data are reported as means ± SEM; n = 8-10 mice per group. Two-way ANOVA: ◦*p* < 0.01, ◦◦◦*p* < 0.001 main effects LPS/vehicle; §*p* < 0.05, §§*p* < 0.01 main effect standard/VTX-enriched diet. Post-hoc: *p < 0.05, **p < 0.01, ***p < 0.001 indicate significant group differences). **Abbreviations:** Never in mitosis gene a (nima)-related expressed kinase 7, NEK7; dorsal hippocampus, DH; ventral hippocampus, VH; frontal-prefrontal cortex, FCx; hypothalamus, HYP; vortioxetine, VTX; lipopolysaccharide, LPS; hours, h.

In the DH, LPS exposure significantly impacted NEK7 expression at the 6 h time-point (F (1,34) = 4.909, *p =* 0.034) while a main effect of the VTX diet was revealed 24 h-post immune stimulation (F (1,32) = 8.527, *p =* 0.007) (Figs. **6A**, **B**). Notably, NEK7 mRNA levels were lower at 24 h in mice fed with a VTX-enriched diet compared to control mice receiving a standard diet (Fig. **6B**).

As for the VH, no main effect and significant differences among the experimental groups were shown at any time point evaluated (Figs. **6C**, **D**).

In the FCx, the immune challenge significantly affected NEK7 expression at the 6 h time-point (F (1,34) = 25.985, *p <* 0.0001) (Figs. **6E**, **F**); in fact, its mRNA levels were significantly increased at 6 h and returned to control levels 24 h after LPS, irrespective of the diet (Fig. **6E**).

In the HYP, two-way ANOVA highlighted the main effect of LPS injection on the gene expression of NEK7 at both time points (6 h: F (1,37) = 21.591, *p <* 0.0001; 24 h: F (1,33) = 49.237, *p <* 0.0001), and of VTX only at 24 h (F (1,33) = 6.143, *p =* 0.019) (Figs. **6G**, **H**). LPS induced a NEK7 upregulation at both time points in animals fed with both standard or VTX-enriched diets (Figs. **6G**, **H**).

## 4 DISCUSSION

In the current study, the expression of inflammasomes, key components in inflammation, within brain areas linked to depression was investigated during systemic inflammation caused by LPS. We found that LPS triggers specific changes in the transcription of these inflammasomes and related subunits across different regions and time points in the brain. Additionally, we discovered that VTX, an antidepressant known for its cognitive benefits in depression, notably counteracts the abnormal NLRP3 inflammasome expression, particularly in the dorsal hippocampus 24 h after LPS exposure, potentially *via* regulating NEK7 expression. The use of LPS-induced *in vivo* mouse models of depression is common in preclinical research to mimic various phenotypic and neuromolecular aspects of depression and explore potential antidepressant drugs [35, 39-46]. For instance, animal models based on LPS-induced NLRP3 inflammasome overactivation are increasingly used, revealing that some antidepressants mitigate the depression-related phenotype *via* NLRP3 modulation [10, 11, 14]. Thus, while most studies have focused on the NLRP3 inflammasome separately in key brain regions, here we aimed to bridge this gap by simultaneously examining multiple inflammasomes and related subunits across these central areas. Our investigation revealed a unique regional profile of inflammasome transcription induced by immune challenges. Notably, we confirmed that 24 h after LPS injection, there was a robust induction of NLRP3 and NLRP1 inflammasomes as well as of ASC and CASP subunits in the brain [22, 47-49]. Additionally, we revealed that some inflammasome genes showed consistent activation across all explored areas while others responded selectively. This diversity in regional response might reflect a specific inflammasome’s role in particular brain regions, possibly driven by the differential sensitivity of microglia cells across these areas [50, 51]. A possible explanation is that the varying activation of inflammasomes triggered by the systemic inflammatory stimulus might be linked to specific cell types, highlighting distinct roles for individual inflammasomes in specific brain regions. Microglia cells predominantly manage inflammasome signaling in the central nervous system. Interestingly, this spatial variation indicates varying sensitivity of microglia to systemic immune triggers across different brain areas [52-58]. Understanding these molecular nuances could be pivotal in developing region-specific therapies targeting inflammasome signaling pathways in depression. Moreover, we explored the time-dependent effects of LPS stimulation at two time points (6 h and 24 h) after injection, revealing varied expression patterns of inflammasome subunits. Some genes responded quickly, while others showed delayed or sustained changes, suggesting potential implications for depression symptoms and treatment responses. More specifically, studying the effects of LPS *in vivo* allowed us to track changes corresponding to the peak sickness response and the onset of depressive symptoms [35], showing variation in the timing of gene activation (*e.g*., NLRP3 and ASC activated early; NLRP1 and CASP1 activated later) [59, 60]. Interestingly, some inflammasome genes exhibited transient changes, while others, like NLRP3 and ASC, remained elevated in specific brain areas for an extended period. This suggests a potential avenue for therapeutics to modulate early and long-term expression of pathogenic inflammasome genes (*e.g*., NLRP3, ASC) with significant clinical implications. Our findings align with the functional differences among inflammatory mediators and emphasize the critical roles of NLRP3 and ASC in inflammasome activation. Notably, with its signal amplification function, ASC reached its peak expression 24 h post-LPS, potentially reflecting its essential role in driving maximal CASP1 activation across various brain regions in response to LPS [61, 62]. In this study, we also explored VTX ability to modulate inflammasomes centrally. This is in light of substantial evidence supporting the role of the NLRP3 inflammasome in depression and cognitive dysfunctions, as well as the ability of different antidepressants to modulate this intracellular system [25, 26, 63]. Investigating its impact on various brain areas critical in depression, such as the DH and FCx involved in cognition [64, 65], and the VH linked to emotional and stress responses like HYP [66], may shed light on its broader therapeutic mechanisms. Dysfunction in these interconnected brain regions can lead to depression, memory issues, emotional dysregulation, and hormonal imbalances due to disrupted integration of cognitive, emotional, and physiological functions [64-66]. Thus, we investigated how VTX influences the NLRP3 inflammasome in specific brain areas. Herein, we confirmed our hypothesis of an area- and time-specific impact of VTX on the NLRP3 inflammasome pathway. Specifically, VTX counteracted the increased gene expression levels of NLRP3 and ASC subunits induced by LPS, particularly in the dorsal hippocampus 24 h post-challenge. This effect aligns with VTX’s observed improvement in memory impairment [35], possibly linked to its modulation of the NLRP3 inflammasome pathway in this brain region. Previous research also supports the role of the dorsal hippocampus in memory, which is consistent with our findings [67-69]. Notably, our observations suggest a significant influence of VTX on this specific molecular pathway, aligning with other studies that show certain antidepressants affecting NLRP3 and ASC expression across the hippocampus to improve depression-like behaviors [70]. Furthermore, we identified the potential role of NEK7 [71] in mediating VTX effect on NLRP3 inflammasome activity [72] in the dorsal hippocampus at 24 h time point. NEK7 is under scrutiny for its regulatory role in NLRP3 activation in various diseases, including depression [28, 36, 71, 73, 74]. While our study expands the understanding of VTX molecular mechanisms [75], future research will need to focus on protein-level variations, downstream effects, and VTX influence on specific cell types, especially microglia. Unravelling these details could pave the way for targeted depression treatments. In conclusion, our study, the first of its kind exploring VTX potential in modulating the NLRP3 inflammasome in a time- and area-specific manner in the brain, could serve as a starting point for further investigations into VTX molecular mechanisms in treating depression and cognitive impairments.

## CONCLUSION

**Fig. (7):**
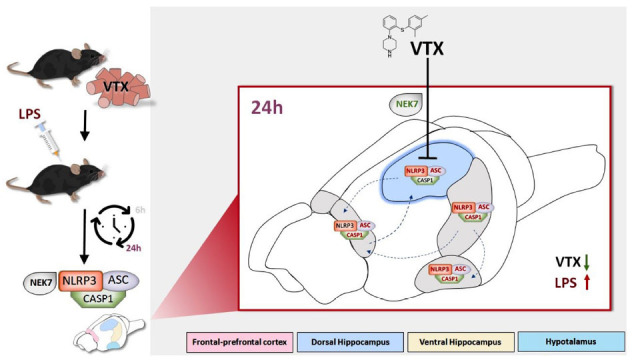
Twenty-four hours post-injection, a chronic pre-treatment with vortioxetine counteracted the increased gene expression levels of NLRP3 and ASC by LPS, particularly in the dorsal hippocampus. Moreover, in the same conditions, vortioxetine downregulated NEK7 expression, possibly contributing to inhibiting NLRP3 activity. Indeed, NEK7 has been implicated in the activation of the NLRP3. Considering that the dorsal hippocampus plays a key role in memory, this effect aligns with VTX observed improvement in memory impairment induced by LPS that we previously observed at the same time points. **Abbreviations:** NLR family, pyrin domain containing 3, NLRP3; apoptosis-associated speck-like protein, ASC; caspase 1, CASP1; never in mitosis gene a (NIMA)-related expressed kinase 7, NEK7; frontal-prefrontal cortex, VTX; lipopolysaccharide, LPS; hours, h.

Vortioxetine countered the increased gene expression levels of NLRP3 and ASC subunits induced by LPS, particularly in the dorsal hippocampus 24 h post-challenge, potentially *via* regulating NEK7 expression (Fig. **7**). Our study, the first of its kind exploring VTX potential in modulating the NLRP3 inflammasome in a time- and area-specific manner in the brain, could serve as a starting point for further investigations into VTX molecular mechanisms in treating depression and cognitive impairments.

## Figures and Tables

**Table 1:** Sequence of each primer used in real‐time PCR.

**Gene**	**NCBI GenBank**	**Sequence**
***NLRP3***	NM_145827.4	FW: AGAAGAGTGGATGGGTTTGCT RV: GCGTTCCTGTCCTTGATAGAG
***ASC***	NM_023258	FW: CAAACGACTAAAGAAGAGTCTG RV: AGAGCTTCCTCATCTTGTCT
***CASP1***	NM_009807.2	FW: CCGTGGAGAGAAACAAGGAG RV: AATGAAAAGTGAGCCCCTGA
***NLRP1***	NM_001004142.2	FW: TGACAAGGGCAGTGACAATC RV: GCCACAAAATGAGGGAGGTA
***AIM2***	NM_001013779.2	FW: CCCAAGCAAAACAAAGTGCG RV: AGTTTTTGGCTTTGCAGCCTT
***NLRC4***	NM_001033367.3	FW: GCTCCTGAACATACCCGACT RV: GTGGTGGTGGTGACAATGAC
***NEK7***	NM_021605	FW: AGCATCCTGTCTCTTGGATGG RV: CACGTGCTTTGGCATCCATT
***CYPA***	NM_008907.2	FW: AGCATACAGGTCCTGGCATC RV: TTCACCTTCCCAAAGACCAC

*Abbreviations: NLR family, pyrin domain containing 3, NLRP3; apoptosis-associated speck-like protein, ASC; caspase 1, CASP1; NLR family pyrin domain containing 1, NLRP1; absent in melanoma 2, AIM2; NLR family, CARD domain containing 4, NLRC4; NIMA related kinase 7, NEK7; cyclophilin A, CypA; forward, FW; reverse, RV.

## Data Availability

Not applicable.

## LIST OF ABBREVIATIONS

AIM2: Absence in Melanoma-2
ASC: Apoptosis-associated Speck-like Protein
CASP1: Caspase-1
CypA: Cyclophilin A
DH: Dorsal Hippocampal Subregion
FCx: Frontal-prefrontal Cortex
HYP: Hypothalamus
LPS: Lipopolysaccharide
MDD: Major Depressive Disorder
NEK 7: Never in Mitosis Gene a (NIMA)-related Expressed Kinase 7
NLRC4: NLR Family CARD Domain Containing 4
NLRP3: NOD-like Receptor Family Pyrin Domain Containing 3
VH: Ventral Hippocampal Subregion
VTX: Vortioxetine

## STANDARDS OF REPORTING

CONSORT guidelines and methodologies were followed.

## References

[1] Liu Q., He H., Yang J., Feng X., Zhao F., Lyu J. (2020). Changes in the global burden of depression from 1990 to 2017: Findings from the Global Burden of Disease study.. J. Psychiatr. Res..

[2] Shorey S., Ng E.D., Wong C.H.J. (2022). Global prevalence of depression and elevated depressive symptoms among adolescents: A systematic review and meta‐analysis.. Br. J. Clin. Psychol..

[3] Culpepper L., Lam R.W., McIntyre R.S. (2017). Cognitive impairment in patients with depression: Awareness, assessment, and management.. J. Clin. Psychiatry.

[4] Pan Z., Park C., Brietzke E., Zuckerman H., Rong C., Mansur R.B., Fus D., Subramaniapillai M., Lee Y., McIntyre R.S. (2019). Cognitive impairment in major depressive disorder.. CNS Spectr..

[5] Varghese S., Frey B.N., Schneider M.A., Kapczinski F., de Azevedo Cardoso T. (2022). Functional and cognitive impairment in the first episode of depression: A systematic review.. Acta Psychiatr. Scand..

[6] Rosenblat J.D., Kakar R., McIntyre R.S. (2016). The cognitive effects of antidepressants in major depressive disorder: A systematic review and meta-analysis of randomized clinical trials.. Int. J. Neuropsychopharmacol..

[7] Kopschina Feltes P., Doorduin J., Klein H.C., Juárez-Orozco L.E., Dierckx R.A.J.O., Moriguchi-Jeckel C.M., de Vries E.F.J. (2017). Anti-inflammatory treatment for major depressive disorder: implications for patients with an elevated immune profile and non-responders to standard antidepressant therapy.. J. Psychopharmacol..

[8] Xia C.Y., Guo Y.X., Lian W.W., Yan Y., Ma B.Z., Cheng Y.C., Xu J.K., He J., Zhang W.K. (2023). The NLRP3 inflammasome in depression: Potential mechanisms and therapies.. Pharmacol. Res..

[9] Roy S., Arif Ansari M., Choudhary K., Singh S. (2023). NLRP3 inflammasome in depression: A review.. Int. Immunopharmacol..

[10] Alcocer-Gómez E., de Miguel M., Casas-Barquero N., Núñez-Vasco J., Sánchez-Alcazar J.A., Fernández-Rodríguez A., Cordero M.D. (2014). NLRP3 inflammasome is activated in mononuclear blood cells from patients with major depressive disorder.. Brain Behav. Immun..

[11] Alcocer-Gómez E., Casas-Barquero N., Williams M.R., Romero-Guillena S.L., Cañadas-Lozano D., Bullón P., Sánchez-Alcazar J.A., Navarro-Pando J.M., Cordero M.D. (2017). Antidepressants induce autophagy dependent-NLRP3-inflammasome inhibition in Major depressive disorder.. Pharmacol. Res..

[12] Guo H., Callaway J.B., Ting J.P.Y. (2015). Inflammasomes: Mechanism of action, role in disease, and therapeutics.. Nat. Med..

[13] Du R.H., Tan J., Sun X.Y., Lu M., Ding J.H., Hu G. (2016). Fluoxetine inhibits NLRP3 inflammasome activation: Implication in depression.. Int. J. Neuropsychopharmacol..

[14] Arioz B.I., Tastan B., Tarakcioglu E., Tufekci K.U., Olcum M., Ersoy N., Bagriyanik A., Genc K., Genc S. (2019). Melatonin attenuates LPS-induced acute depressive-like behaviors and microglial NLRP3 inflammasome activation through the SIRT1/Nrf2 pathway.. Front. Immunol..

[15] Tsai S.J. (2017). Effects of interleukin-1beta polymorphisms on brain function and behavior in healthy and psychiatric disease conditions.. Cytokine Growth Factor Rev..

[16] Alboni S., Cervia D., Sugama S., Conti B. (2010). Interleukin 18 in the CNS.. J. Neuroinflammation.

[17] Milner M.T., Maddugoda M., Götz J., Burgener S.S., Schroder K. (2021). The NLRP3 inflammasome triggers sterile neuroinflammation and Alzheimer’s disease.. Curr. Opin. Immunol..

[18] Panicker N., Kam T.I., Wang H., Neifert S., Chou S.C., Kumar M., Brahmachari S., Jhaldiyal A., Hinkle J.T., Akkentli F., Mao X., Xu E., Karuppagounder S.S., Hsu E.T., Kang S.U., Pletnikova O., Troncoso J., Dawson V.L., Dawson T.M. (2022). Neuronal NLRP3 is a parkin substrate that drives neurodegeneration in Parkinson’s disease.. Neuron.

[19] Voet S., Srinivasan S., Lamkanfi M., van Loo G. (2019). Inflammasomes in neuroinflammatory and neurodegenerative diseases.. EMBO Mol. Med..

[20] Song A.Q., Gao B., Fan J.J., Zhu Y.J., Zhou J., Wang Y.L., Xu L.Z., Wu W.N., Wu W.N. (2020). NLRP1 inflammasome contributes to chronic stress-induced depressive-like behaviors in mice.. J. Neuroinflammation.

[21] Li Y.K., Chen J.G., Wang F. (2021). The emerging roles of absent in melanoma 2 (AIM2) inflammasome in central nervous system disorders.. Neurochem. Int..

[22] Iban-Arias R., Sebastian-Valverde M., Wu H., Lyu W., Wu Q., Simon J., Pasinetti G.M. (2022). Role of polyphenol-derived phenolic acid in mitigation of inflammasome-mediated anxiety and depression.. Biomedicines.

[23] Flores J., Noël A., Fillion M.L., LeBlanc A.C. (2022). Therapeutic potential of Nlrp1 inflammasome, caspase-1, or caspase-6 against alzheimer disease cognitive impairment.. Cell Death Differ..

[24] Li J.M., Liu L.L., Su W.J., Wang B., Zhang T., Zhang Y., Jiang C.L. (2019). Ketamine may exert antidepressant effects via suppressing NLRP3 inflammasome to upregulate AMPA receptors.. Neuropharmacology.

[25] Lee H., Park J.H., Hoe H.S. (2022). Idebenone regulates Aβ and LPS-induced neurogliosis and cognitive function through inhibition of NLRP3 Inflammasome/IL-1β axis activation.. Front. Immunol..

[26] Lonnemann N., Hosseini S., Marchetti C., Skouras D.B., Stefanoni D., Dinarello C.A., Korte M. (2020). The NLRP3 inflammasome inhibitor OLT1177 rescues cognitive impairment in a mouse model of Alzheimer’s disease.. Proc Natl Acad Sci.

[27] Wu X.L., Deng M.Z., Gao Z.J., Dang Y.Y., Li Y.C., Li C.W. (2020). Neferine alleviates memory and cognitive dysfunction in diabetic mice through modulation of the NLRP3 inflammasome pathway and alleviation of endoplasmic-reticulum stress.. Int. Immunopharmacol..

[28] Li P., He Y., Yang Q., Guo H., Li N., Zhang D. (2023). NEK7 inhibition attenuates Aβ42-induced cognitive impairment by regulating TLR4/NF-κB and the NLRP3 inflammasome in mice.. J. Clin. Biochem. Nutr..

[29] Xu Y., Yang Y., Chen X., Jiang D., Zhang F., Guo Y., Hu B., Xu G., Peng S., Wu L., Hu J. (2023). NLRP3 inflammasome in cognitive impairment and pharmacological properties of its inhibitors.. Transl. Neurodegener..

[30] Bruno A., Zoccali R.A., Troili G.M., Scala L., Pandolfo G., Cedro C., Mento C., Santoro V., Spina E., Muscatello M.R.A. (2020). Vortioxetine on cognition in schizophrenia.. J. Clin. Psychopharmacol..

[31] Jeong H.W., Yoon K.H., Lee C.H., Moon Y.S., Kim D.H. (2022). Vortioxetine treatment for depression in alzheimer’s disease: A randomized, double-blind, placebo-controlled study.. Clin. Psychopharmacol. Neurosci..

[32] Nemutlu Samur D., Akçay G., Yıldırım S., Özkan A., Çeker T., Derin N., Tanrıöver G., Aslan M., Ağar A., Özbey G. (2022). Vortioxetine ameliorates motor and cognitive impairments in the rotenone-induced Parkinson’s disease via targeting TLR-2 mediated neuroinflammation.. Neuropharmacology.

[33] Bennabi D., Haffen E., Van Waes V. (2019). Vortioxetine for cognitive enhancement in major depression: From animal models to clinical research.. Front. Psychiatry.

[34] Santos García D., Alonso Losada M.G., Cimas Hernando I., Cabo López I., Yáñez Baña R., Alonso Redondo R., Paz González J.M., Cores Bartolomé C., Feal Painceiras M.J., Íñiguez Alvarado M.C., Labandeira C., García Díaz I. (2022). Vortioxetine improves depressive symptoms and cognition in parkinson’s disease patients with major depression: An open-label prospective study.. Brain Sci..

[35] Alboni S., Benatti C., Colliva C., Radighieri G., Blom J.M.C., Brunello N., Tascedda F. (2021). Vortioxetine prevents lipopolysaccharide-induced memory impairment without inhibiting the initial inflammatory cascade.. Front. Pharmacol..

[36] Liu G., Chen X., Wang Q., Yuan L. (2020). NEK7: A potential therapy target for NLRP3-related diseases.. Biosci. Trends.

[37] Li Y., Abdourahman A., Tamm J.A., Pehrson A.L., Sánchez C., Gulinello M. (2015). Reversal of age-associated cognitive deficits is accompanied by increased plasticity-related gene expression after chronic antidepressant administration in middle-aged mice.. Pharmacol. Biochem. Behav..

[38] Rigillo G., Vilella A., Benatti C., Schaeffer L., Brunello N., Blom J.M.C., Zoli M., Tascedda F. (2018). LPS-induced histone H3 phospho(Ser10)-acetylation(Lys14) regulates neuronal and microglial neuroinflammatory response.. Brain Behav. Immun..

[39] Zakaria R., Wan Yaacob W.M.H., Othman Z., Long I., Ahmad A.H., Al-Rahbi B. (2017). Lipopolysaccharide-induced memory impairment in rats: A model of Alzheimer’s disease.. Physiol. Res..

[40] Cunningham C., Wilcockson D.C., Campion S., Lunnon K., Perry V.H. (2005). Central and systemic endotoxin challenges exacerbate the local inflammatory response and increase neuronal death during chronic neurodegeneration.. J. Neurosci..

[41] Tarr A.J., McLinden K.A., Kranjac D., Kohman R.A., Amaral W., Boehm G.W. (2011). The effects of age on lipopolysaccharide-induced cognitive deficits and interleukin-1β expression.. Behav. Brain Res..

[42] Zhao J., Bi W., Xiao S., Lan X., Cheng X., Zhang J., Lu D., Wei W., Wang Y., Li H., Fu Y., Zhu L. (2019). Neuroinflammation induced by lipopolysaccharide causes cognitive impairment in mice.. Sci. Rep..

[43] Jacewicz M., Czapski G.A., Katkowska I., Strosznajder R.P. (2009). Systemic administration of lipopolysaccharide impairs glutathione redox state and object recognition in male mice.. Folia Neuropathol..

[44] Valero J., Mastrella G., Neiva I., Sánchez S., Malva J.O. (2014). Long-term effects of an acute and systemic administration of LPS on adult neurogenesis and spatial memory.. Front. Neurosci..

[45] Frenois F., Moreau M., O’ Connor J., Lawson M., Micon C., Lestage J., Kelley K.W., Dantzer R., Castanon N. (2007). Lipopolysaccharide induces delayed FosB/DeltaFosB immunostaining within the mouse extended amygdala, hippocampus and hypothalamus, that parallel the expression of depressive-like behavior.. Psychoneuroendocrinology.

[46] O’Connor J.C., Lawson M.A., André C., Moreau M., Lestage J., Castanon N., Kelley K.W., Dantzer R. (2009). Lipopolysaccharide-induced depressive-like behavior is mediated by indoleamine 2,3-dioxygenase activation in mice.. Mol. Psychiatry.

[47] Zhao L.R., Xing R.L., Wang P.M., Zhang N.S., Yin S.J., Li X.C., Zhang L. (2018). NLRP1 and NLRP3 inflammasomes mediate LPS/ATP induced pyroptosis in knee osteoarthritis.. Mol. Med. Rep..

[48] Xie L., Gu Z., Liu H., Jia B., Wang Y., Cao M., Song R., Zhang Z., Bian Y. (2020). The anti-depressive effects of hesperidin and the relative mechanisms based on the NLRP3 inflammatory signaling pathway.. Front. Pharmacol..

[49] Li M.M., Wang X., Chen X.D., Yang H.L., Xu H.S., Zhou P., Gao R., Zhang N., Wang J., Jiang L., Liu N. (2022). Lysosomal dysfunction is associated with NLRP3 inflammasome activation in chronic unpredictable mild stress-induced depressive mice.. Behav. Brain Res..

[50] Silverman H.A., Dancho M., Regnier-Golanov A., Nasim M., Ochani M., Olofsson P.S., Ahmed M., Miller E.J., Chavan S.S., Golanov E., Metz C.N., Tracey K.J., Pavlov V.A. (2014). Brain region-specific alterations in the gene expression of cytokines, immune cell markers and cholinergic system components during peripheral endotoxin-induced inflammation.. Mol. Med..

[51] Jung H., Lee H., Kim D., Cheong E., Hyun Y.M., Yu J.W., Um J.W. (2022). Differential regional vulnerability of the brain to mild neuroinflammation induced by systemic LPS treatment in mice.. J. Inflamm. Res..

[52] de Haas A.H., Boddeke H.W.G.M., Biber K. (2008). Region‐specific expression of immunoregulatory proteins on microglia in the healthy CNS.. Glia.

[53] Grabert K., Michoel T., Karavolos M.H., Clohisey S., Baillie J.K., Stevens M.P., Freeman T.C., Summers K.M., McColl B.W. (2016). Microglial brain region−dependent diversity and selective regional sensitivities to aging.. Nat. Neurosci..

[54] De Biase L.M., Schuebel K.E., Fusfeld Z.H., Jair K., Hawes I.A., Cimbro R., Zhang H.Y., Liu Q.R., Shen H., Xi Z.X., Goldman D., Bonci A. (2017). Local cues establish and maintain region-specific phenotypes of basal ganglia microglia.. Neuron.

[55] Ayata P., Badimon A., Strasburger H.J., Duff M.K., Montgomery S.E., Loh Y.H.E., Ebert A., Pimenova A.A., Ramirez B.R., Chan A.T., Sullivan J.M., Purushothaman I., Scarpa J.R., Goate A.M., Busslinger M., Shen L., Losic B., Schaefer A. (2018). Epigenetic regulation of brain region-specific microglia clearance activity.. Nat. Neurosci..

[56] Furube E., Kawai S., Inagaki H., Takagi S., Miyata S. (2018). Brain region-dependent heterogeneity and dose-dependent difference in transient microglia population increase during lipopolysaccharide-induced inflammation.. Sci. Rep..

[57] Masuda T., Sankowski R., Staszewski O., Prinz M. (2020). Microglia heterogeneity in the single-cell era.. Cell Rep..

[58] Brandi E., Torres-Garcia L., Svanbergsson A., Haikal C., Liu D., Li W., Li J.Y. (2022). Brain region-specific microglial and astrocytic activation in response to systemic lipopolysaccharides exposure.. Front. Aging Neurosci..

[59] Stutz A., Kolbe C.C., Stahl R., Horvath G.L., Franklin B.S., van Ray O., Brinkschulte R., Geyer M., Meissner F., Latz E. (2017). NLRP3 inflammasome assembly is regulated by phosphorylation of the pyrin domain.. J. Exp. Med..

[60] Franklin B.S., Bossaller L., De Nardo D., Ratter J.M., Stutz A., Engels G., Brenker C., Nordhoff M., Mirandola S.R., Al-Amoudi A., Mangan M.S., Zimmer S., Monks B.G., Fricke M., Schmidt R.E., Espevik T., Jones B., Jarnicki A.G., Hansbro P.M., Busto P., Marshak-Rothstein A., Hornemann S., Aguzzi A., Kastenmüller W., Latz E. (2014). The adaptor ASC has extracellular and ‘prionoid’ activities that propagate inflammation.. Nat. Immunol..

[61] Dick M.S., Sborgi L., Rühl S., Hiller S., Broz P. (2016). ASC filament formation serves as a signal amplification mechanism for inflammasomes.. Nat. Commun..

[62] Nagar A., Rahman T., Harton J.A. (2021). The ASC speck and NLRP3 inflammasome function are spatially and temporally distinct.. Front. Immunol..

[63] Lyu D., Wang F., Zhang M., Yang W., Huang H., Huang Q., Wu C., Qian N., Wang M., Zhang H., Zheng S., Chen J., Fu Y., Zhang C., Li Z., Hong W. (2022). Ketamine induces rapid antidepressant effects via the autophagy-NLRP3 inflammasome pathway.. Psychopharmacology.

[64] Lee I., Kesner R.P. (2003). Time-dependent relationship between the dorsal hippocampus and the prefrontal cortex in spatial memory.. J. Neurosci..

[65] Yavas E., Gonzalez S., Fanselow M.S. (2019). Interactions between the hippocampus, prefrontal cortex, and amygdala support complex learning and memory.. F1000 Res..

[66] Jimenez J.C., Su K., Goldberg A.R., Luna V.M., Biane J.S., Ordek G., Zhou P., Ong S.K., Wright M.A., Zweifel L., Paninski L., Hen R., Kheirbek M.A. (2018). Anxiety cells in a hippocampal-hypothalamic circuit.. Neuron.

[67] Moser M-B., Moser E.I. (1998). Functional differentiation in the hippocampus.. Hippocampus.

[68] Sannino S., Russo F., Torromino G., Pendolino V., Calabresi P., De Leonibus E. (2012). Role of the dorsal hippocampus in object memory load.. Learn. Mem..

[69] Gálvez-Márquez D.K., Salgado-Ménez M., Moreno-Castilla P., Rodríguez-Durán L., Escobar M.L., Tecuapetla F., Bermudez-Rattoni F. (2022). Spatial contextual recognition memory updating is modulated by dopamine release in the dorsal hippocampus from the locus coeruleus.. Proc. Natl. Acad. Sci..

[70] Trojan E., Chamera K., Bryniarska N., Kotarska K., Leśkiewicz M., Regulska M., Basta-Kaim A. (2019). Role of chronic administration of antidepressant drugs in the prenatal stress-evoked inflammatory response in the brain of adult offspring rats: Involvement of the NLRP3 inflammasome-related pathway.. Mol. Neurobiol..

[71] Zhao N., Li C., Di B., Xu L. (2020). Recent advances in the NEK7-licensed NLRP3 inflammasome activation: Mechanisms, role in diseases and related inhibitors.. J. Autoimmun..

[72] Schmacke N.A., Gaidt M.M., Szymanska I., O’duill F., Stafford C.A., Chauhan D., Fröhlich A.L., Nagl D., Pinci F., Schmid-Burgk J.L., Hornung V. (2019). Priming enables a NEK7-independent route of NLRP3 activation.. bioRxiv.

[73] Liang L., Wang H., Hu Y., Bian H., Xiao L., Wang G. (2022). Oridonin relieves depressive‐like behaviors by inhibiting neuroinflammation and autophagy impairment in rats subjected to chronic unpredictable mild stress.. Phytother. Res..

[74] Fang Z.E., Wang Y., Bian S., Qin S., Zhao H., Wen J., Liu T., Ren L., Li Q., Shi W., Zhao J., Yang H., Peng R., Wang Q., Bai Z., Xu G. (2024). Helenine blocks NLRP3 activation by disrupting the NEK7-NLRP3 interaction and ameliorates inflammatory diseases.. Phytomedicine.

[75] Rigillo G., Ciani M., Benatti C., Blom J.M.C., Tascedda F., Pani L., Alboni S., Brunello N. (2023). Vortioxetine attenuates neuroinflammation by modulating the NOD-like receptor family pyrin domain containing 3 inflammasome activation in microglia: implications for cognitive function.. Neurosci. Appl..

